# Cyto-, myelo- and chemoarchitecture of the prefrontal cortex of the *Cebus *monkey

**DOI:** 10.1186/1471-2202-12-6

**Published:** 2011-01-13

**Authors:** Roelf J Cruz-Rizzolo, Miguel AX De Lima, Edilson Ervolino, José A de Oliveira, Claudio A Casatti

**Affiliations:** 1 1Campus de Araçatuba, UNESP - Univ Estadual Paulista, Departamento de Ciências Básicas, São Paulo, Brazil

## Abstract

**Background:**

According to several lines of evidence, the great expansion observed in the primate prefrontal cortex (PfC) was accompanied by the emergence of new cortical areas during phylogenetic development. As a consequence, the structural heterogeneity noted in this region of the primate frontal lobe has been associated with diverse behavioral and cognitive functions described in human and non-human primates. A substantial part of this evidence was obtained using Old World monkeys as experimental model; while the PfC of New World monkeys has been poorly studied.

In this study, the architecture of the PfC in five capuchin monkeys (*Cebus apella*) was analyzed based on four different architectonic tools, Nissl and myelin staining, histochemistry using the lectin *Wisteria floribunda *agglutinin and immunohistochemistry using SMI-32 antibody.

**Results:**

Twenty-two architectonic areas in the *Cebus *PfC were distinguished: areas 8v, 8d, 9d, 12l, 45, 46v, 46d, 46vr and 46dr in the lateral PfC; areas 11l, 11m, 12o, 13l, 13m, 13i, 14r and 14c in the orbitofrontal cortex, with areas 14r and 14c occupying the ventromedial corner; areas 32r, 32c, 25 and 9m in the medial PfC, and area 10 in the frontal pole. This number is significantly higher than the four cytoarchitectonic areas previously recognized in the same species. However, the number and distribution of these areas in *Cebus *were to a large extent similar to those described in Old World monkeys PfC in more recent studies.

**Conclusions:**

The present parcellation of the *Cebus *PfC considerably modifies the scheme initially proposed for this species but is in line with previous studies on Old World monkeys. Thus, it was observed that the remarkable anatomical similarity between the brains of genera *Macaca *and *Cebus *may extend to architectonic aspects. Since monkeys of both genera evolved independently over a long period of time facing different environmental pressures, the similarities in the architectonic maps of PfC in both genera are issues of interest. However, additional data about the connectivity and function of the *Cebus *PfC are necessary to evaluate the possibility of potential homologies or parallelisms.

## Background

Several studies carried out in different contexts and based on different theoretical premises indicate that the great expansion observed in the primate prefrontal cortex (PfC) was accompanied by the emergence of new cortical areas during phylogenetic development [1-5]. As a consequence of this process, this region of the primate frontal lobe was converted into a structurally and functionally heterogeneous area. The primate PfC can be initially divided into lateral, medial and orbital surfaces and further subdivided into areas with distinct architectonic and connectional characteristics. This heterogeneity may explain the variety of behavioral alterations and the diversity and specificity of cognitive deficits observed in human and non-human primates after lesions or reversible suppression of restricted areas of the PfC [6-18].

**Figure 1:**
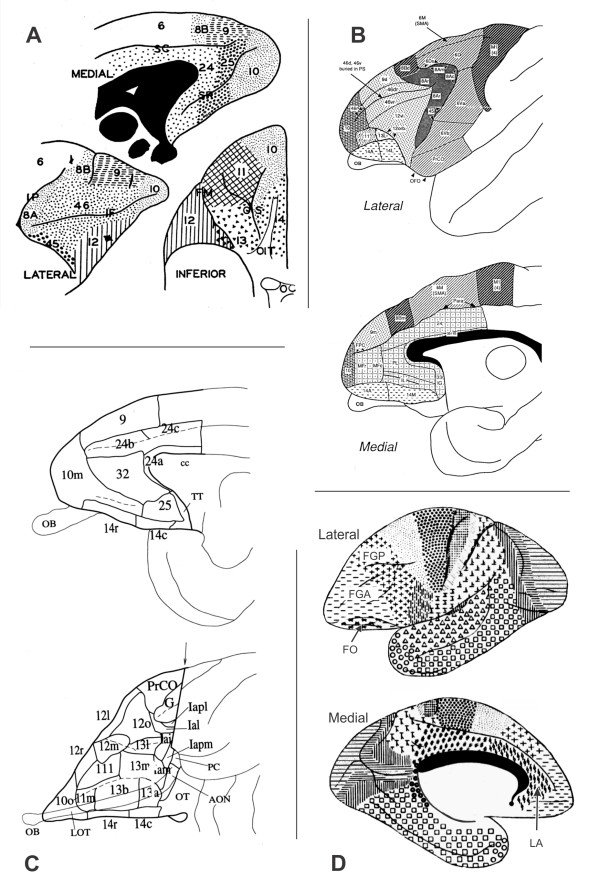
**Architectonic maps of monkey PfC, taken from four different studies**. **A**, the widely cited cytoarchitectonic map of *Macaca *PfC by Walker (1940). In **B **and **C **maps from more recent studies of *Macaca *PfC by Carmichael and Price (1994) and Preuss and Goldman-Rakic (1991), respectively. **D**, from von Bonin (1938). In this parcellation the *Cebus *PfC was subdivided into three areas, FGP, frontalis granularis posterior; FGA, frontalis granularis anterior; FO, frontal orbital area and limbic anterior area, LA, in medial surface.

Architectonic studies of primate PfC confirm this heterogeneity. In Old World monkeys, Brodmann [1] divided the PfC into six different areas. Subsequently, Vogt and Vogt [19] differentiated nine areas in the *Cercopithecus *dorsolateral PfC (DlPfC). In 1940, Walker [20] carried out a specific study on the rhesus PfC (*Macaca mulatta*), in an attempt to adapt his observations to the patterns noted by Brodmann [21] in the human brain. Walker [20] defined nine cytoarchitectonic areas in the rhesus PfC (Figure 1A) which would be comparable to areas of similar nomenclature in the human brain. This cytoarchitectonic division proposed by Walker is the most universally accepted. However, subsequent studies carried out in different contexts and using connectional, cyto-, myelo- and chemoarchitectonic techniques (Figure 1B,C) have modified this initial parcellation of the monkey PfC either by the subdivision of pre-existing areas or by the modification of their limits [5,22-29].

All of these studies were carried out in Old World monkeys, whereas the PfC of New World monkeys has been poorly studied. The evolutionary history of this group of primates is still unclear and subject to disagreement [30] but it is accepted that they have evolved independently from Old World monkeys over a period of 35 million years. The effect of this parallel evolution on the organization of phylogenetically recent cortical areas such as those of the PfC still needs to be elucidated.

**Figure 2:**
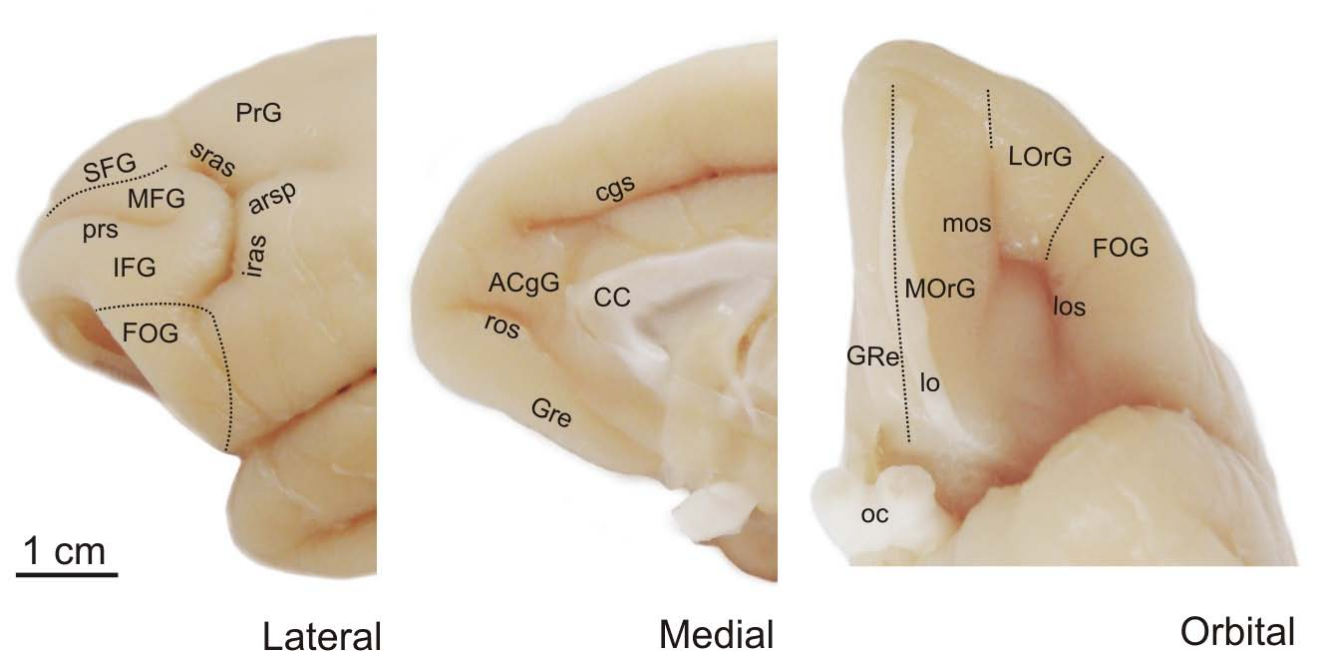
**Surface view of the lateral, medial and orbital prefrontal cortex of *Cebus apella*, showing the anatomical division adopted in this study**. Dotted lines define approximate borders between gyris and solid lines indicate the sulci.

The capuchin monkey (*Cebus apella*) was chosen for this study due to its similarity with the most intensively studied *Macaca *monkey. *Cebus *exhibits brain and body sizes comparable with those of several species of macaque monkeys, reducing possible allometric differences. In addition, the pattern of cortical fissuration is virtually identical in *Cebus *and *Macaca*, facilitating anatomical comparison. Unlike other New World monkeys commonly used in brain research, such as squirrel monkeys and marmosets, the *Cebus *PfC is the only one that consistently exhibits a well-defined arcuate sulcus in the frontal lobe separated from and arching around the caudal end of the principal sulcus (prs; Figure 2), an anatomical configuration that some authors consider as one criterion that distinguishes cercopithecoids from ceboids [31]. Although this anatomical similarity raises the possibility of potential homologies or parallelisms, the remarkable lack of more consistent data about the architecture, connectivity and function of the *Cebus *PfC prevents any progress in this issue.

The only study on the architecture of the *Cebus *PfC, carried out in the context of an overall analysis of the entire cerebral cortex, distinguished it in four different areas (Figure 1D). The *Cebus *PfC parcellation proposed by von Bonin [32] differs considerably from the macaque parcellation proposed by Walker [20] (Figure 1A), a fact that may indicate great architectonic differences in the PfC of these two species.

In view of the limitation of von Bonin's study, such as the use of a single animal and only Nissl staining, a more comprehensive architectonic study of the *Cebus *PfC is necessary to evaluate possible architectonic similarities and differences between *Cebus *and *Macaca*. In the present study, we used the traditional Nissl and myelin staining methods besides histochemistry to lectin *Wisteria floribunda *agglutinin and immunohistochemistry to SMI-32 antibody, two architectonic tools widely employed in the demarcation of cortical and subcortical morphofunctional areas of several species.

## Results

**Figure 3:**
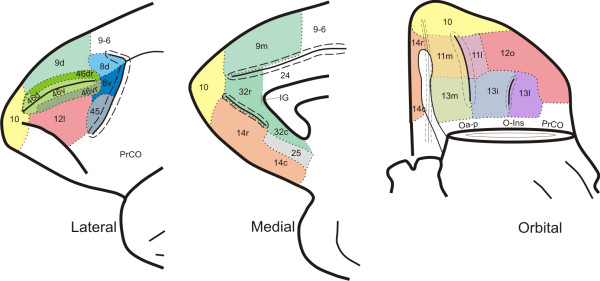
**Surface view of the lateral, medial and orbital prefrontal cortex of *Cebus apella*, with the architectonic parcellation based on results of the present study**. Dotted lines define approximate architectonic borders; solid lines indicate fundus of sulci, and dashed lines define lip or angulus of sulcus. In orbital view, temporal pole has been cut off to expose posterior orbital surface.

**Figure 4:**
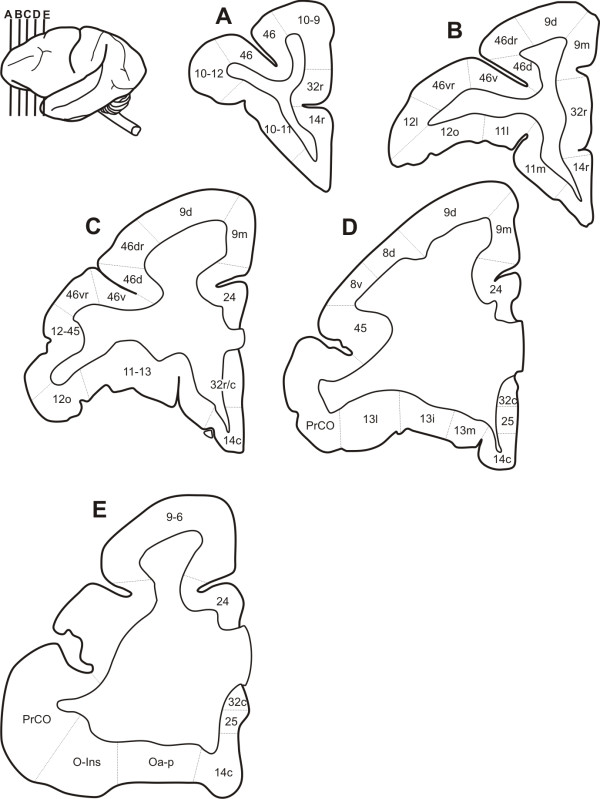
**Coronal sections showing areal borders**. Drawings of coronal sections through five different rostrocaudal levels of *Cebus *left hemisphere, showing areal borders.

In this study, twenty-two areas were differentiated in the *Cebus *PfC (Figures 3; 4). Considering the cortical similarity observed between *Macaca *and *Cebus*, each area was designated by the same numeric terminology adopted in previous studies carried out in Old World monkeys, which follow the architectonic scheme used by Walker [20] (Figure 1B). This terminology was adopted not to establish homologies but rather to permit a rapid topographic comparison due to the widespread acceptance of the division proposed by Walker for the primate PfC.

### External morphology of the PfC in *Cebus *monkeys

The pattern of cortical fissuration of the *Cebus *brain has been addressed by several authors emphasizing its great similarity with the macaque brain [33,34]. The external anatomical aspect of the *Cebus *PfC is illustrated in Figure 2.

Following the criteria adopted in previous studies carried out in monkeys, the *Cebus *PfC was divided into three regions: lateral, medial (MPfC) and orbital (orbitofrontal cortex, OfC). The lateral region extends from the frontal pole to the arcuate sulcus, including the dorsolateral PfC and part of the ventrolateral convexity. Although in the initial description of von Bonin [32] the caudal limit of "area frontalis granularis" of *Cebus *extends caudally in relation to the arcuate sulcus (Figure 1D), it was observed that this sulcus established a limit between the agranular-dysgranular cortex of the precentral gyrus (PrG) and the granular cortex of the prefrontal area.

The MPfC occupies the medial surface of the PfC from the frontal pole to the anterior extremity of the cingulate sulcus (cgs). However, since architectonic studies of PfC in macaques include the precallosal extension of the anterior cingulate gyrus (ACgG), this area was also included in the present study. Finally, the OfC occupies the ventral surface of the PfC extending from the frontal pole rostrally to the anterior perforated substance.

### Overview of staining patterns

#### Nissl

**Figure 5:**
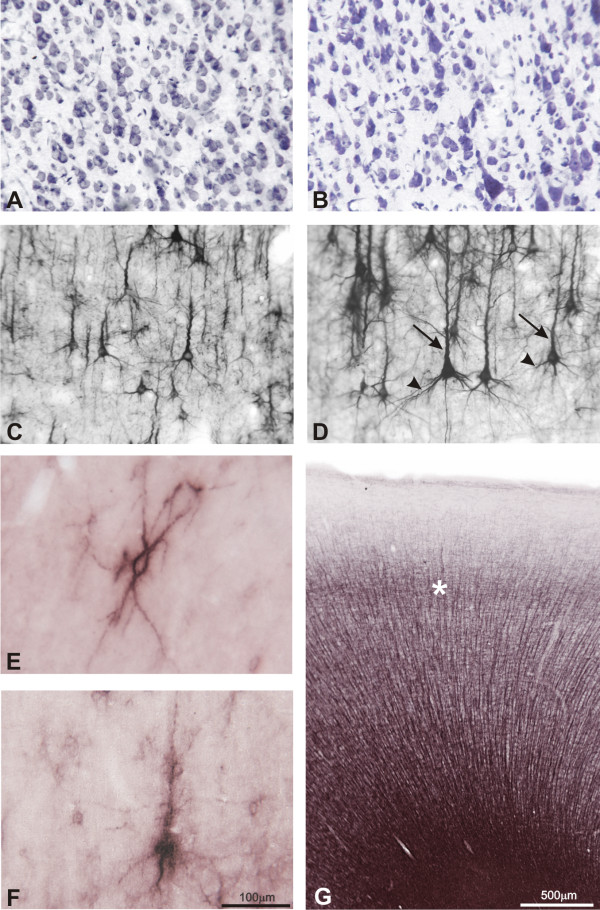
**High magnification photomicrographs showing cellular details of techniques used in this study**. In **A **and **B**, photomicrographs taken from layer V of Nissl stained sections. Small, medium, and large-sized pyramidal neurons can be observed. In **C **and **D**, cell bodies and dendrites of pyramidal cells showing SMI-32 immunoreactivity in cortical layers III (**C**) and V (**D**). Note intense staining in cell bodies, apical (arrows) and basal (arrowheads) dendrites. In **E **perineuronal nets (PNs) stained with WFA ensheath layer III non-pyramidal neurons in area 45, and in **F **PNs surrounding layer V pyramidal neurons in area 32. In all cell types, staining intensity decreases from perikaryon to distal portions of dendrites. In **G**, myelin staining of area 9m. Note thick vertical fascicles and outer band of Baillarger (asterisk). Calibration bar in **F **applies to all figures except **G**

The cytoarchitecture of the *Cebus *PfC (and the frontal lobe as a whole) revealed a granular - dysgranular - agranular rostrocaudal gradation. An example of this transition could be observed in the superior frontal gyrus (SFG), occupied by areas 10 and 9d. Caudally, layer IV gradually narrowed, disappearing in the precentral gyrus (PrG). This type of cortex, bordering the agranular cortex, characterized by a rudimentary layer IV with no clear laminar demarcation is designated dysgranular, and represents a transition between the granular and agranular isocortex. In the lateral surface of the PfC, areas 10, 12l, 46v, 46d, 46vr, 46dr, 8v, 8d, and 45 had granular characteristics, with well developed layers II and IV, clearly demarcated from adjacent laminae. Although a few subtle cytoarchitectonic differences had been observed in these areas, the border between them was not always noted using this staining method. A similar transition was observed in the medial and orbital surfaces of the *Cebus *PfC (Figure 5A,B).

#### WFA

The plant lectin *Wisteria floribunda *agglutinin (WFA) labels N-acetylgalactosamine residues of the extracellular matrix. Areas with intense WFA staining differed from faintly stained areas by the density and intensity of perineuronal nets (PNs) and by the different intensity of the neuropil. The cortical labeling was arranged in bands that could occupy one or more layers. Generally, infragranular layers showed the densest staining in each area, with the labeling occasionally reaching the white matter. In some areas, layers II and III were also labelled, although less intensely than infragranular layers. Nets were observed surrounding the soma and proximal segment of the axon and dendrites of non-pyramidal and some pyramidal neurons mostly distributed in layers V and VI (Figure 5E,F). An overall rostral to caudal labeling gradient was observed, with the agranular and dysgranular regions of the caudal PfC showing the densest WFA labeling.

#### SMI-32

SMI-32 exhibited a heterogeneous labeling pattern across the *Cebus *PfC. Two bands with varying levels of SMI-32 immunoreactivity were usually observed over layers III and V. These bands which were designated supra and infragranular bands showed immunoreactivity present in small to large pyramidal neurons, including their proximal processes and fragments of apical dendrites (Figure 5C,D).

In the brain sections examined in this study, the greatest density of SMI-32 positive neuronal soma was noted in supragranular layers, mainly in layer IIIc, and some in layer IV. Comparatively, few immunoreactive neuronal soma were observed in infragranular layers. In addition, a variable level of neuropil immunoreactivity both in the supra- and infragranular bands was observed.

#### Myelin

The black-gold staining pattern distinguished densely myelinated areas in the lateral PfC from less stained areas in the medial and orbital surfaces. In addition to this basic characteristic, in some areas the visualization of vertical fascicles or the inner and outer bands of Baillarger, (ibB and obB) allowed to establish areal boundaries (Figure 5G).

### Architectonic parcellation

#### Lateral PfC (Table 1,)

#### Area 10

##### Nissl

**Figure 6:**
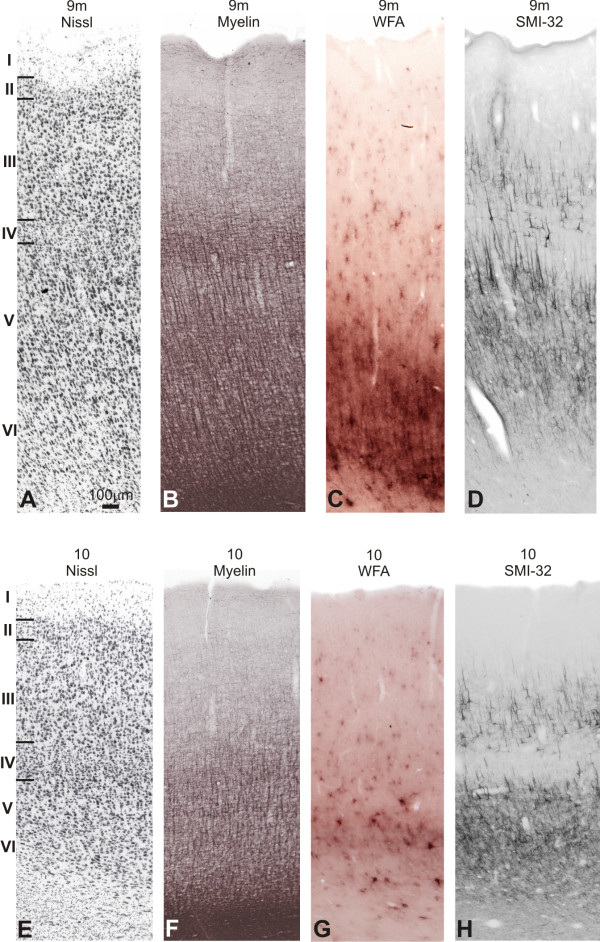
**Semi-adjacent sections showing laminar organization and staining pattern of areas 9 m and 10**. In **A **and **E**, Nissl; **B **and **F**, myelin-staining; **C **and **G**, WFA labeling; and **D **and **H**, SMI-32 immunostaining. Roman numerals in Nissl stained sections indicate cortical layers. Calibration bar in **A **applies to all figures.

The frontopolar region had a well developed layer II. Layer III contained small-sized cells with weak stain, except in IIIc, where they were more stained and larger. Layer IV was well developed. Cells in Va were more densely packed than in IIIc, and Vb almost blended with layer VI where small-sized neurons predominated (Figure 6E).

##### WFA

This area was not sharply demarcated in relation to the adjoining caudal areas using this technique (Figure 6G). It exhibited a weaker WFA staining pattern than that observed in area 9. Supragranular layers exhibited discrete pale nets, and the neuropil was weakly stained. The labeling was somewhat more intense in layers V-VI.

##### SMI-32

The supragranular band consisted of weak neuropil labeling, profiles of apical dendrites and soma of sparsely distributed pyramidal neurons. The neuropil in the infragranular band was more densely labeled, exhibiting few immunoreactive neurons in layer Va (Figure 6H).

##### Myelin

The frontal pole revealed poor to moderate myelination, basically concentrated in infragranular layers, where thin vertical fibers extended from the white matter (Figure 6F). The SFG had moderate myelination, becoming more intense caudalwards.

#### Area 9

This area occupied part of the lateral (area 9d) and medial (area 9m) surfaces of the superior frontal gyrus (SFG). On the DlPfC, 9d ventrally reached the border between the SFG and the medial frontal gyrus (MFG); and on the medial surface area 9m extended up to the cingulate sulcus (Figures 3; 4). It was limited caudally by the cortex of the PrG but this transition could not be sharply demarcated.

##### Nissl

**Figure 7:**
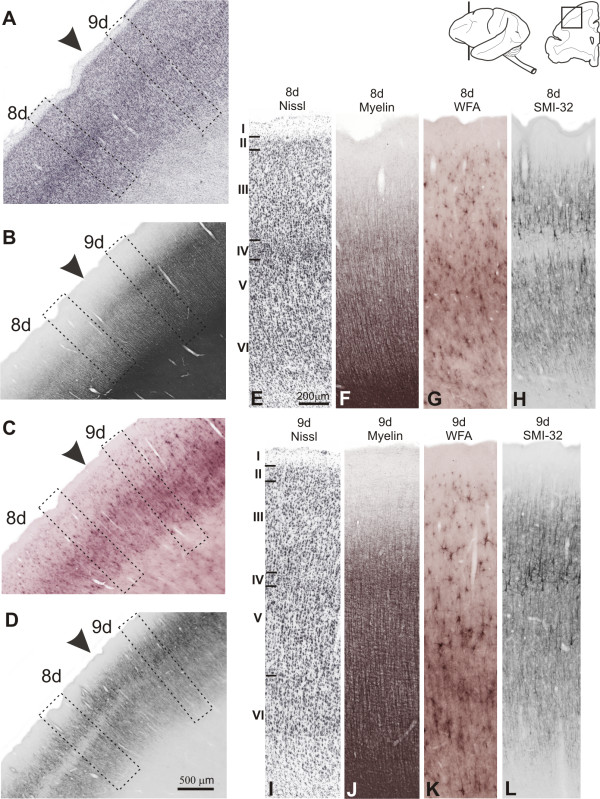
**Architectonic transition in SFG of *Cebus *monkey**. On the left column, low-power photomicrograph of semi-adjacent coronal sections comparing Nissl (**A**), myelin staining (**B**), WFA-labeling (**C**) and SMI-32 immunostaining (**D**), showing transition (arrowhead) between areas 9d-8d in DlPfC. Boxes on photomicrographs indicate the location of higher magnication views of these areas (**E **to **L**). Schematic drawing of *Cebus *brain in upper right side of picture shows level and location of sections **A**, **B**, **C **and **D**.

In this area, layer II was not well developed. Layer IIIa contained small-sized cells, sparsely scattered with weak to moderate stain. Layers IIIb and IIIc had small and medium-sized cells, respectively. Cells of IIIc were slightly more stained and separated from layer Va by a poorly developed layer IV. Layer Va exhibited well pigmented medium-sized cells and layers Vb and VI had small-sized cells and no clear limits (Figure 7A,I). Radial striations were observed in the infragranular layers reaching layer III. This architectonic pattern can also be observed in 9 m (Figure 6A).

##### WFA

In 9d, the most intensely stained band coincided with layer VI, reaching the white matter (Figure 7C,K). This band exhibited numerous nets surrounding non-pyramidal and a few pyramidal neurons, besides the neuropil being densely stained, decreasing in layer V. Layer V showed nets involving small- and medium-sized cells and the staining could also be observed surrounding vertical fibers that occasionally reached layer III. In IIIc the neuropil is faintly stained, but some nets could still be observed. In the medial extension of this area (9m), the staining intensity in layer VI increased although the labeling in supragranular layers was weaker. In addition, the labeling of vertical fibers was denser than that observed in the dorsal surface (Figure 6C).

##### SMI-32

**Figure 8:**
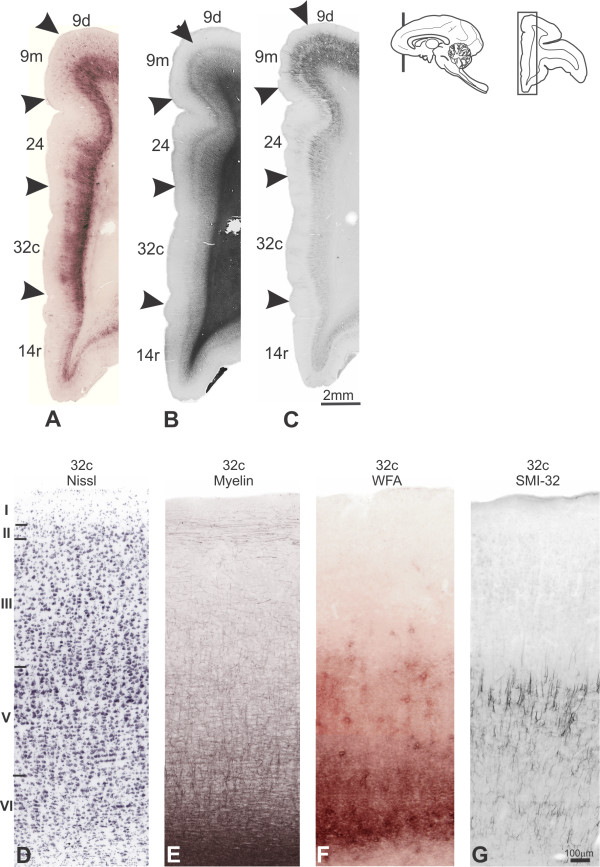
**Architectonic parcellation of medial PfC cortex in *Cebus *monkeys**. **A**, **B **and **C **shown low-power photomicrograph of semi-adjacent coronal sections comparing WFA-labeling (**A**), myelin-staining (**B**) and SMI-32 immunostaining (**C**), indicating approximate boundaries between architectonic areas in medial PfC (arrowheads). **D**, **E**, **F **and **G **shown high-power photomicrographs of area 32c stained by Nissl, myelin, WFA and SMI-32 methods. Schematic drawing of *Cebus *brain in upper left side of picture shows level and location of sections **A**, **B **and **C**.

Caudally, area 9d exhibited denser immunoreactivity than area 10. The bilaminar pattern was less evident; and there was an intense labeling of neuropil and processes. Several small- to medium-sized dense immunoreactive pyramidal neurons were observed in layers IIIc, IIIb and IV. In the infragranular layers, the number of immunoreactive neurons was small and the labeling was restricted mainly to neuropil and fragments of apical dendrites (Figure 7D,L). The labeling of 9m was similar to 9d, although less intense (Figures 6D; 8C).

##### Myelin

In 9d, infragranular layers were heavily myelinated, with prominent vertically oriented fiber bundles extending from the white matter to layer III. The obB was easily discernible and supragranular layers exhibited a sparse plexus of fine myelinated fibers (Figure 7B,J). The medial extension of the SFG (area 9m) showed similar staining pattern, but supragranular layers were more myelinated and obB more evident than in 9d (Figures 6B; 8B).

#### Periprincipalis areas (46d, 46dr, 46vr and 46v)

Following the nomenclature adopted by Walker [20], the periprincipalis region was designated area 46. However, in the present study this region was subdivided into four different architectonic sectors: 46d and 46v in the dorsal and ventral walls of the prs respectively, and areas 46dr and 46vr in the dorsal and ventral crowns.

##### Nissl

**Figure 9:**
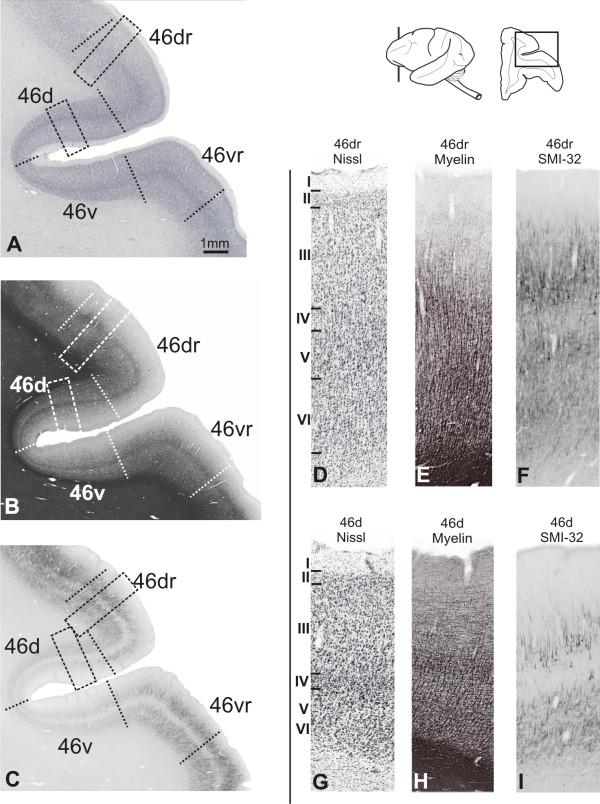
**Architectonic parcellation of periprincipalis region in *Cebus *monkeys**. On left column, low-power photomicrograph of semi-adjacent coronal sections comparing Nissl (**A**), myelin-staining (**B**) and SMI-32 immunostaining (**C**), showing approximate boundaries between architectonic areas (dashed lines) in dorsolateral PfC. Boxes on photomicrographs indicate location of high-power photomicrographs of areas 46dr and 46d shown on right. Schematic drawing of *Cebus *brain in upper right side of picture shows level and location of **A**, **B **and **C **sections.

**Figure 10:**
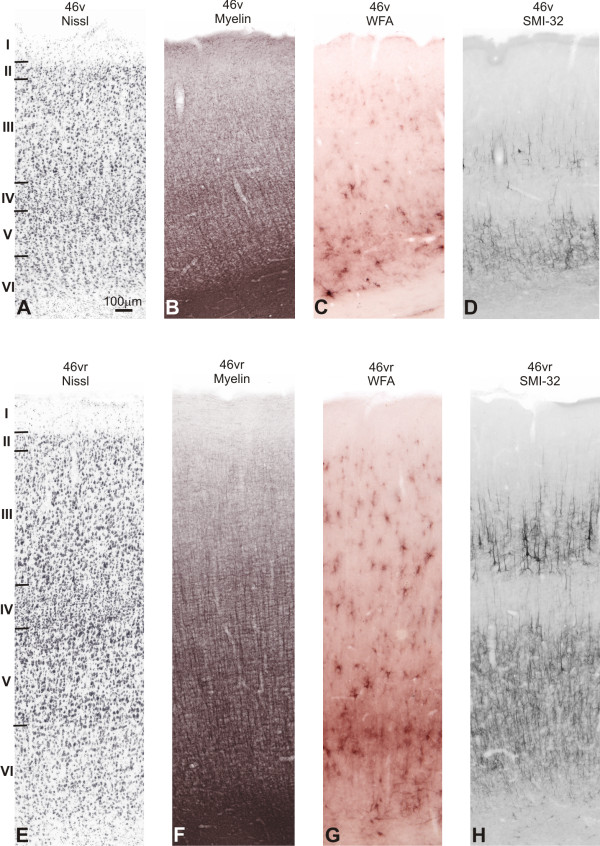
**Semi-adjacent sections showing laminar organization and staining pattern of areas 46v and 46vr**. In **A **and **E**, Nissl; **B **and **F**, myelin-staining; **C **and **G**, WFA labeling; and **D **and **H**, SMI-32 immunostaining. Roman numerals in Nissl stained sections indicate cortical layers. Calibration bar in **A **applies to all figures.

In the banks of prs, area 46d exhibited a well developed and densely packed layer II, showing clear limits with layer III. Layer IIIa had small-sized neurons, moderately stained. Neurons in IIIc layer were small- to medium-sized and intensely stained. Layer IV was well developed and in Va neurons were intensely stained. The limit between layers Vb and VI was not clear, because both had medium-sized cells and moderate pigmentation (Figure 9A,G). In 46v, the architectonic characteristics were similar, but pyramidal neurons in layer III were less densely packed than in 46d. In these areas (46d and 46v) supragranular layers (II and III) were more developed than infragranular layers (Figures 9A; 10A).

In the dorsal crown of prs, area 46dr exhibited transitional characteristics between areas 46d and 9d. The most distinctive aspect was the density decrease in layers II and IV dorsalwards. Va exhibited medium-sized cells slightly more stained than in IIIc. Layers Vb and VI had pale stained small-sized cells, with no clear definition between both layers (Figure 9A,D).

In the ventral crown of prs, area 46vr exhibited similar characteristics to 46v but with layer IV somewhat more developed, showing densely packed cells. Layer III presented clear lamination and the supra- and infragranular compartments were equally prominent. Radial striations could be noted in the infragranular layers, mainly in layer V (Figures 9A; 10E).

##### WFA

The staining in 46dr was weaker than in area 9d. Discrete nets surrounding small cells with the neuropil weakly stained were observed in supragranular layers. Deep layers had a staining pattern similar to 9d, but somewhat less intense. In the caudal half of the prs, the labeling was more intense, but rostrally it was weak, with no clear demarcation with the adjacent area 10.

The walls of the prs exhibited lower levels of WFA reactivity. In the dorsal bank (46d), a small number of nets involved non-pyramidal neurons in layers III and IV, and a faintly stained band of neuropil with some darkly stained nets were present in deeper layers. In the ventral bank (46v) the WFA staining was still weaker (Figure 10C).

WFA staining increased in the ventral crown of the prs (46vr; Figure 10G). The most intensely stained band coincided with layer V, with moderately labeled neuropil and a high concentration of nets mainly encircling non-pyramidal neurons. In layer VI the labeling was a bit weaker. Superficial layers had poorly stained neuropil, with nets surrounding small and medium-sized non-pyramidal cells.

##### SMI-32

Dorsally, the immunoreactivity in area 46dr pattern was less intense and showed clear-cut limits with area 9d (Figure 9C,F). Small- to medium-sized dense immunoreactive pyramidal neurons were observed in the supragranular band, especially in layer IIIc and sparsely in layer V. The lips of the principal sulcus were slightly immunoreactive, clearly distinguishing this region (areas 46d and 46v) from neighboring areas 46dr and 46vr (Figure 9C). In the upper lip (area 46d), immunoreactive neuronal structures were discrete in relation to area 46dr, occupying only layer III and forming occasional clusters (Figure 9I). The infragranular band contained only neuropil and apical dendrite profiles. Immunoreactivity was less pronounced in area 46v than in area 46d, exhibiting discrete soma immunoreactivity (Figures 9C,I; 10D).

Area 46vr showed a significant increase in the SMI-32 immunoreactivity, permitting a clear distinction with area 46v (Figure 9C). Layers IIIb-IIIc had many small- to medium-sized pyramidal neurons. The infragranular band consisted essentially of neuropil, neuronal processes and some pyramidal cells.

##### Myelin

Area 46dr exhibited lighter myelination than 9d. The obB was narrower and less stained, and vertical fibers were more sparse and thinner (Figure 9B,E). Myelination increased in area 46d. The supragranular layers displayed delicate oBe, constituted by thin horizontal fibers. These characteristics were also observed in 46v, but here the supragranular layers showed lower levels of myelin staining (Figures 9B,H; 10B).

In 46vr, infragranular layers were more heavily myelinated than in area 46v with evident vertical fiber fascicles, however the obB was not clearly discernible. The supragranular layers were poorly myelinated (Figures 9B; 10F).

#### Area 12

This area occupied part of the ventrolateral convexity of the lateral PfC (area 12l), reaching the orbital surface of the fronto-orbital gyrus (FOG; area 12o).

##### Nissl

**Figure 11:**
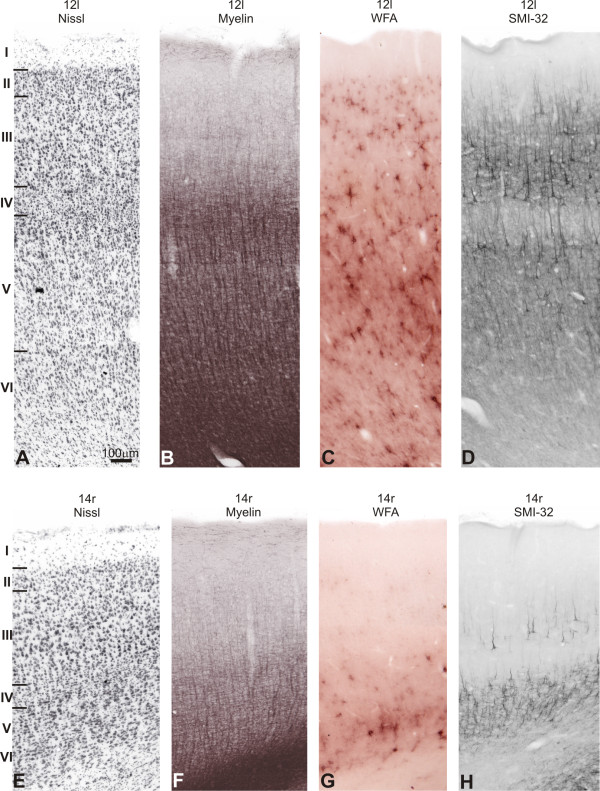
**Semi-adjacent sections showing laminar organization and staining pattern of areas 12l and 14r**. In **A **and **E**, Nissl; **B **and **F**, myelin-staining; **C **and **G**, WFA labeling; and **D **and **H**, SMI-32 immunostaining. Roman numerals in Nissl stained sections indicate cortical layers. Calibration bar in **A **applies to all figures.

In the ventrolateral convexity, layer IV seemed narrower in 12l than in 46vr, and the lamination in layer III was less evident. There was no obvious predominance between supra- and infragranular layers. Caudally, some darkly stained cells could be distinguished in layers IIIc and Va, similar to the adjoining area 45 (Figure 11A). In 12o, layer IV was narrower than in 12l and cells in IIIc were somewhat larger and more stained. Supragranular layers were more prominent than the infragranular ones.

##### WFA

The cortex in area 12 was more intensely stained than the adjacent cortical areas 45 and 46vr. In 12l, the most intensely labeled band coincided with layer V, reaching layer VI. The neuropil was intensely labeled and a high concentration of nets could be observed (Figure 11C). Layers III and IV exhibited a band of WFA staining with nets surrounding medium-sized neurons and weakly stained neuropil. On the orbital surface, the staining pattern of 12o was similar, but the labelling of layers III-IV was discrete. Caudally, the emergence of the precentral opercular cortex (PrCO) in the ventral PrG caused a variation in the WFA staining. WFA labeling was more intense than that observed in area 12, concentrating on layers V-VI and reaching the white matter.

##### SMI-32

**Figure 12:**
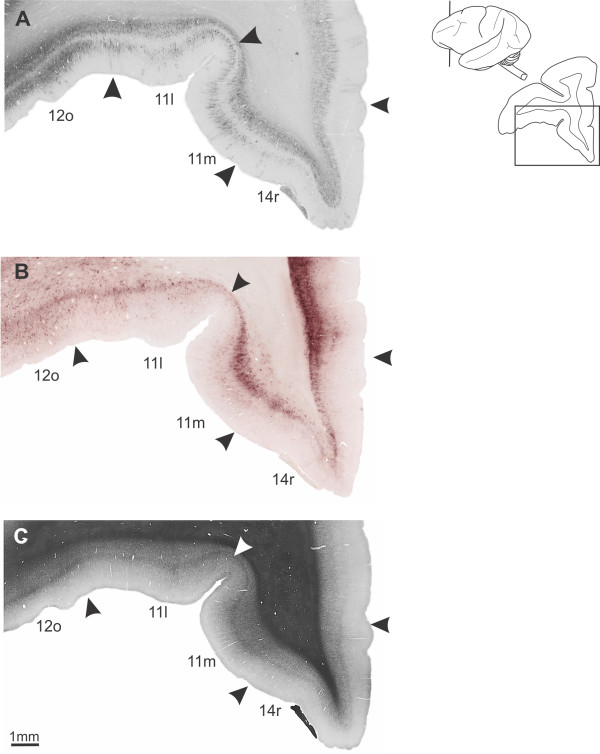
**Architectonic parcellation of rostral orbitofrontal cortex in *Cebus *monkeys**. **A**, **B **and **C **shown low-power photomicrograph of semi-adjacent coronal sections comparing SMI-32 immunostaining (**A**), WFA-labeling (**B**) and myelin-staining (**C**), indicating approximate boundaries between architectonic areas in orbital anterior PfC (arrowheads). Schematic drawing of *Cebus *brain in upper right side of picture shows level and location of sections **A**, **B **and **C**.

Ventrally, the labeling pattern in area 12l was denser than in area 46vr, increasing the number of immunoreactive neurons in the supra and infragranular bands (Figure 11D). Area 12o had immunoreactive characteristics similar to 12l but somewhat less intense. The bilamination was clear, with numerous pyramidal neurons both in the supragranular and infragranular bands, although with the greatest number in layer III (Figure 12A).

##### Myelin

Area 12l exhibited stronger myelination than area 46vr, with evident obB and heavy staining in the infragranular layers (Figure 11B). Area 12o showed similar staining pattern but somewhat less intense than 12l.

#### Prearcuate areas (45, 8d and 8v)

Technical artifacts due to sulcus presence and plane-of-section problems impaired a clear analysis of the prearcuate region, near the caudal end of the prs. Area 45 occupied the anterior bank of the inferior arm of the arcuate sulcus, extending anteriorly to the caudal third of the inferior frontal gyrus (IFG; Figures 3; 4). Dorsally, still in the anterior bank of the arcuate sulcus areas 8d and 8v were distinguished.

##### Nissl

area 45 exhibited granular layers II and IV very well developed and clear limit between layers II and III. In IIIc, large-sized and darkly-stained pyramidal cells gave this area a peculiar characteristic. These large and well-stained cells were also observed in Va. Vb and VI showed small-sized cells.

In 8v granular layers II and IV were well developed. In IIIa and IIIb cells were small, sparsely packed and with low staining. In contrast to area 45, IIIc and Va displayed medium-sized cells, somewhat more stained in Va. Layers Vb and VI had poorly stained small-sized cells. The cytoarchitectonic pattern in 8d was similar, but cells in layer IV were somewhat more sparsely distributed. Radial striations were observed both in 8d and 8v (Figure 7A,E).

##### WFA

Area 45 demonstrated a large number of strongly stained nets and moderately stained neuropil in layer IV, reaching layers III and Va, besides a less stained band in layer VI. Between these two bands there were a few nets and the neuropil was discretely stained. Dorsally, the labeling in 8d was weaker than that observed in area 45, with only one band of neuropil being visible in layer V and a few nets moderately stained in layers V and III (Figure 7G). In 8v, the neuropil in layer IV was somewhat more intense than in 8d.

##### SMI-32

Area 45 had moderate labeling level. The supragranular band contained soma profiles surrounded by intense neuropil. There was also an increase of neuropil labeling in layer IV, but the bilaminar aspect remained. The infragranular band exhibited a moderate neuropil labeling and scarce dendritic profiles. Dorsally, area 8d had a moderate level of immunoreactivity, with medium- to small-sized pyramidal cells in layer IIIc, and in a lesser degree in layers IV, IIIa and V (Figure 7H). Ventrally, in 8V the labeling was similar, but with a somewhat denser neuropil.

##### Myelin

Myelination increased ventrally in the anterior bank of the *as*. Area 8 was not clearly subdivided and exhibited well myelinated obB with thin vertical fibers extending from the white matter (Figure 7F). Superficial layers were poorly myelinated with a fine fiber plexus. Area 45 revealed a heavy myelination pattern in deep cortical layers although without clear organization of vertical fibers. Superficial layers were moderately myelinated in this area, with a fine plexus of sparsely distributed fibers.

**Table 1:** Architectonic characteristics of the lateral PfC

	Nissl	WFA	SMI-32	Myelin
10	Well developed granular layers. Small-sized cells in layer III and more densely packed cells in Va. Vb almost blended with layer VI where small-sized neurons predominated.	Very lightly stained. Labeling concentrates in V-VI.	Moderate immunostaining. SG band with weak neuropil labeling, some fragments of apical dendrites and somas sparsely distributed. Neuropil in IG band is more densely stained and a few somas in Va.	Poor to moderate myelination, with thin vertical fibers in IG layers.

9d	Cells in IIIc more stained and separated from layer Va by a poorly developed layer IV. Layers Vb and VI have small-sized cells and no clear limits. Vertical striations in V and IIIc.	Intensely stained band in VI and a fainter band in Va with some vertical fibers. A few nets in IIIc	Intensely stained. Many cells and processes deeply stained in SG band and a few in IG band.	Moderate to intense myelination. IG layers with vertical fibers. Evident oBb. Sparse fine fibers plexus in SG layers

46dr	Granular. Cells are more densely packed in layer V than in III. Cellular density in layers II and IV decreases dorsally.	Moderately stained. A few small nets with poorly stained neuropil in SG layers. Staining in IG layers similar to 9d, but less intense.	Moderately stained. Clear-cut boundaries with 9d. Bilaminar aspect. Small- to medium-sized pyramidal neurons intensely stained in IIIc, and very few in V.	Lighter myelination than 9d. oBb is narrower and less stained, and vertical fibers are more sparse and thinner.

46d	Granular. Well developed and densely packed layer II. Medium-sized cells intensely stained in Va. SG layers more prominent than IG layers.	Very lightly stained. Faintly stained band of neuropil with some dark nets in IG layers. Very few nets in III and IV.	Lightly stained. Clear limits with 46dr. Very weak neuropil staining and a few clusters of cellular bodies in III. Neuropil and dendrite fragments in IG band	Moderate myelination. Faint iBe and sparse thin vertical fibers in IG layers. Sparse plexus and horizontal fibers in SG layers.

46v	Similar to 46d. No limits between layers V and VI.	Similar to 46d, but weaker.	Weaker than in 46d. almost no immunoreactive somas can be observed	Similar to 46d

46vr	Granular. Layer IV more developed and densely packed than in 46v. Clear lamination in III. SG and IG layers equally prominent. Radial striations in V.	Moderately stained. Good neuropil labeling in Vb, with high concentration of nets. Labeling weaker in VI. SG layers poorly stained, with some sparse nets.	Moderately stained. Clear-cut boundaries with 46v. Immunoreactive cells in IIIb-IIIc. IG band with diffuse neuropil, neuronal processes and a few stained cells.	Moderate myelination. IG layers more heavily myelinated than 46v, with well-stained vertical fibers. oBb less evident. SG layers poorly myelinated.

12l	Granular. Layer IV narrower than in 46vr, and lamination in III less evident. No obvious predominance between SG and IG layers. Caudally, darkly-stained cells in IIIc and Va.	Intensely stained. Denser band in VI, with neuropil and many nets reaching layer VI. Paler band in IV-III with nets and poorly stained neuropil.	Intensely stained. Many stained neurons in V-VI, but a higher number in the SG band. Bilaminar pattern.	Moderate to intense myelination. Evident oBb and heavy staining in IG layers.

8d	Granular. In IIIc and Va cells are of medium size and darkly stained. Layers Vb and VI have cells of small size and are less stained.	Lightly stained, with a band of neuropil in IV and a few moderately stained nets in IV and III.	Moderately stained, with medium to small stained cells in IIIc, and in a lesser degree in IV, IIIa and V.	Distinct oBb and thin vertical fibers. SG layers poorly myelinated.

8v	Similar to 8d, but cells in layer IV more densely packed.	Similar to 8d, but with darker neuropil staining in IV.	Similar to 8d but with a denser staining of neuropil.	Similar to 8d

45	Granular. Large-sized and darkly stained pyramidal cells In IIIc and Va.	Moderately stained. Band with dense nets in IV, reaching III and Va. Moderately stained neuropil in VI. Faint band in Vb.	Moderately stained. Bilaminar aspect. Cell body fragments and intense neuropil in SG band. Pale neuropil in IV. Moderate labeling (neuropil and fragments) in IG band.	Moderate myelination (heavy myelination in IG layers). SG layers poorly myelinated.

### OfC and gyrus rectus (Table 2)

#### Area 11

##### Nissl

On the orbital surface, area 11 m exhibited a thin and sparse layer II. Layer III was also sparse and contains small- to medium-sized pyramidal neurons with some densely stained neurons in IIIc. The limit between layers IIIc and IV was well-defined. Neurons in Va were somewhat more densely packed. Layers Vb and VI had small-sized neurons and the limits between layers were not visible. In 11l, layer IV was narrow and Va showed well stained medium-sized cells, a characteristic that differentiates this area from the adjoining 12o.

##### WFA

Area 11l (Figure 12B) exhibited a band in layer V, reaching layer VI and the white matter. In this band, darkly stained nets were observed surrounding neuronal soma and horizontal fibers, and the neuropil labeling was moderately stained. In IV we observed small nets and weakly stained neuropil, besides pale nets in layer III. In the mos, this arrangement gradually disappeared, and labeling was almost absent in the parafundic cortex. Medially, 11 m showed a compact band in layer VI, with moderately stained neuropil and darkly stained nets involving medium-sized cells. In the remaining layers, the labeling was almost absent.

##### SMI-32

There was a clear decrease in the density of SMI-32 staining on the orbital surface. 11l had clear-cut limits with its neighbouring area 12o (Figure 12A). This area exhibited a faint labeling of neuropil in the supra and infragranular bands. Small densely stained pyramidal neurons could be observed in layer III, and rarely in infragranular layers. Medially, 11 m still preserved bilaminar characteristics. Layer III contained immunoreactive neurons and moderate immunoreactive neuropil with a broader infragranular band (Figure 12A).

##### Myelin

Area 11l had sparse myelination, with obB and infragranular layers less stained than area 12o. In 11m, the obB was faintly stained and ibB was not discernible. Vertical fiber fascicles were observed in infragranular layers in this area, resembling the aspect observed in 46vr (Figure 12C).

#### Area 13

##### Nissl

Following Walker's parcellation of *Macaca *PfC [20], the present study designated the central orbital region of *Cebus *monkey as area 13. However, due to the architectonic heterogeneity, it was further subdivided into lateral (13l), intermediary (13i) and medial (13m) areas. In 13m, layer II was thin and compact, without clear limits with IIIa. Layer IIIb was well-developed, constituted by a discrete cluster of small-sized neurons; while IIIc exhibited moderately stained small- to medium-sized neurons. In Va, neurons had medium-sized and were more stained than in IIIc, and Vb blended with layer VI.

Comparatively with 13m, 13i exhibited less developed layers II and IV. Layers Vb and VI had moderately stained small-sized neurons. These cytoarchitectonic characteristics could also be observed in 13l, however, this area contained larger cells in Va.

##### WFA

**Figure 13:**
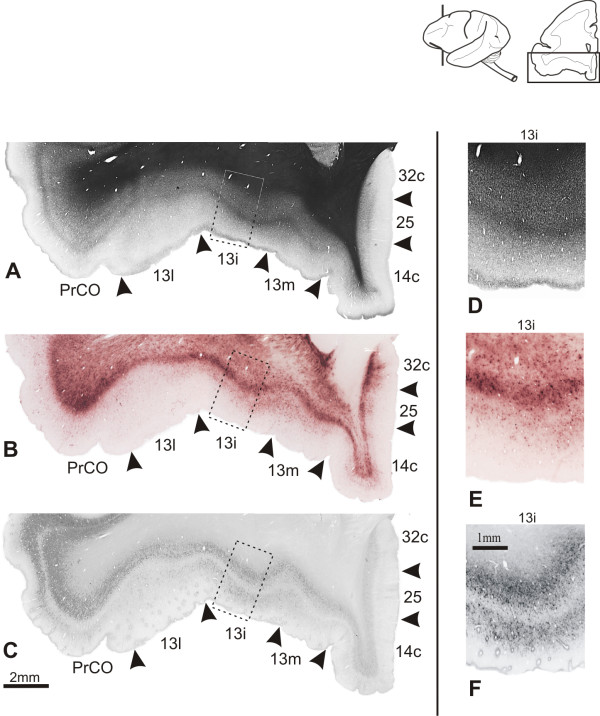
**Architectonic parcellation of the caudal orbitofrontal cortex in *Cebus *monkeys**. On left column, low-power photomicrograph of semi-adjacent coronal sections comparing myelin-staining (**A**), WFA-labeling (**B**) and SMI-32 immunostaining (**C**), showing approximate boundaries between architectonic areas in caudal OfC (arrowheads). Boxes on photomicrographs indicate location of higher magnication views of area 13i showed in **D**, **E **and **F**. Schematic drawing of *Cebus *brain in upper right side of picture shows level and location of sections **A**, **B **and **C**.

Staining becomes more intense caudally in the central orbital region. The 13i was the most intensely stained, showing a bilaminar aspect. The superficial band was weakly stained, with sparse labeling of neuropil and pale nets involving small non-pyramidal neurons. This superficial band was almost indiscernible in the adjoining areas 13l and 13 m (Figure 13B). The deep band occupied the lower part of layer V, with a well-stained neuropil and larger and more abundant nets than observed in the superficial band. This deep band reached layer VI and the white matter, extending over the orbital extension of the claustrum. WFA staining decreased medially. In 13 m the WFA labeling pattern was narrow, being mainly observed in layer VI (Figure 13B,E).

##### SMI-32

Among the subdivisions of area 13, only 13i had a bilaminar pattern (Figure 13C,F). The staining intensity in the infragranular band was similar in the three subdivisions, with neuropil and fragments of cellular bodies without clear pyramidal shape. The supragranular band exhibited moderate immunoreactivity only in 13i with discrete neurons and processes distributed in layer III.

##### Myelin

In the central orbital region, area 13i had light to moderate myelination, but stronger than the adjoining cortical areas 13l and 13m. The obB was clearly discernible in 13i and a fine fiber plexus could be observed in supragranular layers. Deep layers had moderate staining. In 13l and 13 m the staining was lighter and more diffuse, with short vertical fiber bundles extending from the white matter to 13 m (Figure 13A,D).

#### Area 14

##### Nissl

In the anterior part of the gyrus rectus (GRe), area 14r had layer II poorly developed with no clear limits with layer III. Layers IIIa and IIIb had sparse small-sized cells with moderate staining. In IIIc, cells were slightly more stained and larger than IIIb. Layer IV was not well-developed and small-sized neurons predominate in infragranular layers. The limit between layers Vb and VI was not clear (Figure 11E). Caudally, 14c exhibited cytoarchitecture similar to area 14r, although decreasing the cellular density in granular layers II and IV and radial striations in infragranular layers.

##### WFA

The WFA staining considerably decreased in the GRe. At the anterior level, area 14r was not sharply labeled from the adjacent areas 11l and 10 (Figures 8A; 11G). The most important characteristic that differentiates area 14 from the laterally located area 11 was absence of labeling in supragranular layers and the less stained white matter. The labeling concentrated in a narrow band over layer VI, with pale nets and moderately stained neuropil. Caudally (14c), the labeling was weaker and WFA involved thin vertical fibers in deeper layers (Figure 13B).

##### SMI-32

In the GRe, the staining intensity was extremely weak. Rostrally (14r) the infragranular band had modest immunostaining, corresponding primarily to the neuropil and a few perikarya, and very few immunoreactive somas in layer IIIc. Caudally, 14c exhibited very light immunostaining, limited to neuropil in the infragranular band (Figures 8C; 11H; 12A; 13C).

##### Myelin

The GRe (area 14r) had lower levels of myelin staining than neighbouring areas. Only some short and thin vertical fibers emerging from the white matter were present but did not reach supragranular layers (Figure 12C). Caudally, 14c was still less myelinated (Figures 8B; 11F; 13A).

**Table 2:** Architectonic characteristics of orbital PfC

	Nissl	WFA	SMI-32	Myelin
12o	Granular. Layer IV narrower than in 12l. Well stained cells in IIIc. SG layers more prominent than IG.	Intensely stained. Similar to 12l but band in III-IV not discernible.	Intensely stained. Clear bilamination. Many cells in SG and IG bands, with greatest number in III.	Well myelinated. Similar but less intense than 12l.

11l	Granular. Limit between II and IIIa is less evident than in 11m, and layer IV is somewhat narrow. Well stained medium-sized cells in Va.	Moderately stained. Band with moderate neuropil and deeply stained PNs in V reaching layer VI and white matter. In IV small PNs. Very pale PNs in III.	Moderately stained. Clear-cut boundaries with area 12o. Moderate labeling of neuropil and dendritic fragments in SG and IG bands. Small densely stained cell bodies in III and some in IV.	Sparse myelination. OBb and IG layers less stained than 12o

11m	Granular. Thin and sparse layer II. Medium-sized cells with intense pigmentation in IIIc. Densely packed neurons in Va. No clear limits between V and VI.	Lightly stained. Compact and dense band in VI, with moderately stained neuropil and dense PNs.	Discernible bilamination. SG band more stained than in 11l, with cells and processes in III. IG band has moderate staining of neuropil and fragments, but no somas.	Sparse myelination. OBb is faintly stained. Fascicles of vertical fibers in IG layers.

13i	Granular - dysgranular rostrocaudal transition. Layers Vb and VI have moderately stained small-sized neurons.	Moderately stained, increasing caudally. SG band weakly stained, with sparse neuropil and pale PNs. Well stained band in V, reaching VI and white matter.	Moderately stained, decreasing caudally. Bilaminar pattern. IG band with neuropil and fragments. Moderate immunoreactivity in SG band with very few somas and processes in III.	Light to moderate myelination, although stronger than in 13l and 13m. Evident OBb. Fine plexus in SG layers. Moderate staining in IG layers.

13l	Similar to 13i but cells somewhat bigger in Va.	Slightly stained, increasing caudally. No visible superficial band. Narrow band in layer VI.	Moderately stained. IG band similar to 13i. Much lower level of staining in SG layers. No visible bilaminar pattern.	Staining is lighter and more diffuse than in 13i.

13m	Granular - dysgranular rostrocaudal transition. Well developed layer IIIb with sparsely packed small-sized neurons. Cells more stained in IIIc and Va. Vb blends with layer VI.	Poorly stained, increasing caudally.	Similar to 13l.	Similar to 13l but with short vertical fibers in IG layers.

14r	In IIIc and Va cells are larger and more stained than in IIIa-b. No clear limit between Vb and VI.	Poorly stained. No clear-cut limits with areas 11 and 10. Narrow band over layer VI, with pale nets and moderately stained neuropil.	Faintly stained band in IG layers, with neuropil and fragments, and a few somas.	Poorly myelinated. No obvious horizontal bands. Some short and thin vertical fibers in IG layers.

14c	Similar to 14r, but with very faint layers II and IV. Radial striations in IG layers.	Poorly stained. Thin vertical fibers in deep layers.	Similar to 14r, but lighter.	Poorly myelinated.

### Medial PfC (Table 3)

As the dorsal, anterior and ventral borders of this surface represent medial extensions of areas already described, we will describe its central portion.

#### Areas 32 and 25

##### NIssl

**Figure 14:**
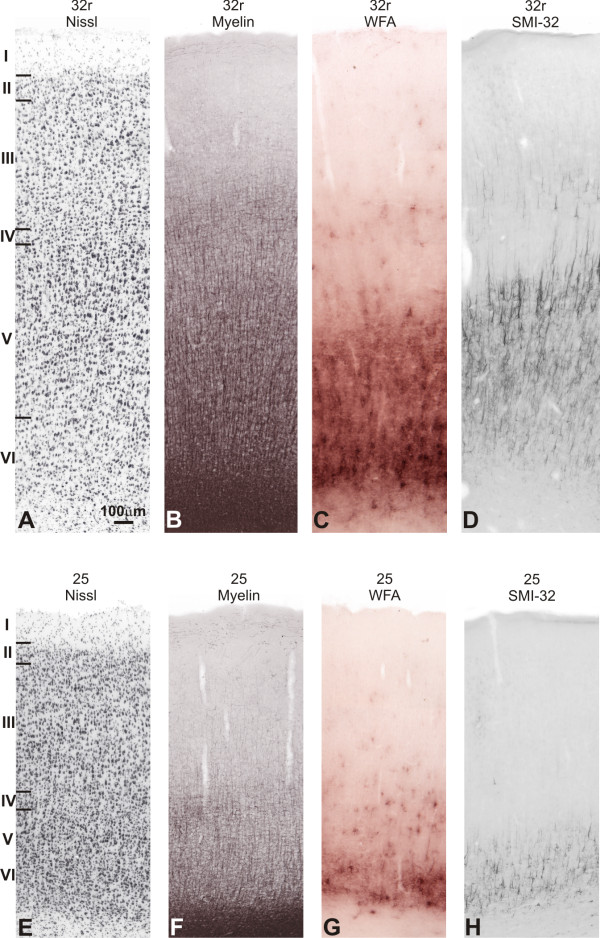
**Semi-adjacent sections showing laminar organization and staining pattern of areas 32r and 25**. In **A **and **E**, Nissl; **B **and **F**, myelin-staining; **C **and **G**, WFA labeling; and **D **and **H**, SMI-32 immunostaining. Roman numerals in Nissl stained sections indicate cortical layers. Calibration bar in **A **applies to all figures.

In the medial surface, area 32 occupied most part of the ACgG. Area 32c was situated anteriorly to the corpus callosum and circumscribed dorsally and ventrally by the cgs and rostral sulcus, respectively. Infragranular layers were more developed in relation to the supragranular compartment. Layer II was poorly developed and had no clear limits with the densely packed layer III. There was no obvious lamination in layer III, while layer IV was absent. Layer V had radial striations and small- to medium-sized cells, which were somewhat larger and more stained than in layer III. There was no clear limit between layers V and VI (Figure 8D). Rostrally, area 32r showed an overall larger cortical thickness than 32c. A thin and cell-sparse layer IV could be visualized. Layer Va contained well-stained medium-sized cells, contrasting with small-sized cells observed in layer VI (Figure 14A).

Ventrally to the rostrum of the corpus callosum, area 25 showed supragranular layers more developed than area 32c, and there was an evident subdivision between layers II and III. Layer III had sparse clusters of small-sized neurons with moderate staining, no clear lamination and scarcely discernible layer IV. In layer V, almost blending with layer VI, cells were slightly larger and more densely packed than in layer III (Figure 14E).

##### WFA

Areas in the medial surface were sharply labeled with WFA (Figure 8A). Area 32c exhibited a narrow but very intense WFA band in layer VI, reaching layer V. Patches of deeply stained neuropil nearly prevented the visualization of nets that, when visible, revealed strong labeling pattern (Figures 8A,F; 13B). Rostrally, 32r had similar labeling pattern, however the band over infragranular layers was wider, exhibiting some very pale nets reaching layer III (Figure 14C).

Area 25 could be differentiated from the adjacent dorsal area 32c and ventral area 14c by clear-cut boundaries (Figures 13B; 14G). The labeling was weak and only a narrow band over layer VI reaching layer V could be observed.

##### SMI-32

Area 32c had discrete immunoreactivity. It was observed only a sparse immunoreactive band of neuropil in infragranular layers with very few pyramidal neurons and dendrites concentrated in layer V. Rostrally, the labeling in 32r was somewhat more intense and some pyramidal neurons could also be seen in layer IIIc. The labeling in 25 was similar to that observed in area 32c, but still lighter (Figures 8G; 14D,H).

##### Myelin

On the medial surface, myelination considerably decreased caudalwards. Area 32c exhibited moderate to poor staining pattern (Figure 8B,E). Thin and short vertical fibers extending from the white matter were observed, but rarely targeted superficial layers. The labeling in 32r was also weak, but obB and ibB were still discernible (Figure 14B). Ventrally, area 25 was poorly myelinated, but somewhat more stained than area 32c (Figures 13A; 14F).

**Table 3:** Architectonic characteristics of medial PfC

	Nissl	WFA	SMI-32	Myelin
32c	Agranular. IG predominates over SG compartment. Medium-sized cells in Va and smaller in III. Layer V blends with VI.	Narrow but very intense band in layer VI, reaching layer V, with patches of strongly labeled PNs.	Weak immunoreactivity. Band in IG layers with sparse neuropil and very few somas and dendrites.	Lightly myelinated. No obvious horizontal bands.

32r	A thin and cell-sparse layer IV can be visualized.	Similar to 32c, but the labeled band over IG layers is wider, with some pale nets reaching layer III.	Immunoreactivity somewhat more intense than 32c, with some pyramidal neurons in layer IIIc.	Moderate myelination. Discernible oBb and iBb. Thin and short vertical fibers in IG layers.

25	Layer IV scarcely discernible. SG layers somewhat more developed than in 32c. Clear limits between II and III. Small-sized cells sparsely packed with no clear lamination in III. Layer V almost blends with layer VI.	Poorly stained. Clear-cut limits with the adjacent dorsal 32c and ventral 14c. Narrow band over layer VI reaching layer V, with a few nets and dense neuropil near white matter.	Similar to 32c, but still lighter.	Poorly myelinated, but somewhat more stained than 32c.

## Discussion

The parcellation of the *Cebus *PfC adopted in this study considerably modified the scheme initially proposed by von Bonin [32] for this species (Figure 1D) but is in line with previous studies on Old World monkeys [5,20,24-29]. Thus, the remarkable anatomical similarity observed between the brains of genera *Macaca *and *Cebus *may extend to architectonic aspects.

### Comparison with previous architectonic maps of the monkey PfC

In this study, twenty-two different areas were differentiated in the *Cebus *PfC (Figures 3; 4). This number is greater than that previously recognized by von Bonin [32] in the same species using cytoarchitectonic criteria (Figure 1D). Due to the different approaches used in the two studies, it is difficult to compare the current results with those obtained by von Bonin and even by other authors who have used only cytoarchitectonic techniques. In fact, the inherent subjectivity related with cytoarchitectonic observations has led to different interpretations concerning the limits between areas and the criteria defining them. To avoid this ambiguity, the combination of several architectonic tools allowed more direct and reproducible denition of the extent and boundaries of areas in the PfC.

Two other factors should be mentioned when analyzing the differences between the present findings and those reported by von Bonin. First, von Bonin's observations were based on a single animal. Second, the criteria used by von Bonin and his collaborators to divide cytoarchitectonic areas were more restricted than those used by others, justifying their criticism of the division proposed by Walker [20] for the *Macaca *PfC [22]. More recent studies, however, using different architectonic methods, connectional and physiological data have confirmed the existence of a larger number of areas in the primate PfC, corroborating Walker's initial observations [5,23-29,35-39].

In the lateral surface of the *Cebus *PfC, while von Bonin [32] (Figure 1D) differentiated only two areas, the posterior and anterior "area frontalis granularis", eleven areas were differentiated in the current study: 9d, 8(d and v), 45, 46 (d, dr, v, vr), 12l, and 10, topographically comparable with the homonymous areas described for *Macaca *by Walker [20]. There are, however, some differences between the present observations and those of Walker.

In the *Macaca genus*, there is disagreement on the description of the prearcuate region. Walker [20] recognized area 8A in the anterior bank of the superior ramus of the arcuate sulcus (sras), area 8B extending dorsally in the SFG, and area 45 occupying part of the inferior ramus of the arcuate sulcus (iras), extending rostrally. While more recent studies confirm these findings [5,28], others confined area 8 to the prearcuate region [25]. Recent analysis of area 45 also differed from the division initially proposed by Walker. Using architectonic and connectional criteria, Petrides and Pandya [29] designated the area which lies in the ventral part of the rostral bank of the lower limb of the arcuate sulcus as 45B, and its rostral extension as 45A. This division was also used by Gerbella et al. [39], who provided a detailed description of the architectonic organization of the caudal ventrolateral PfC of the macaque monkey, including part of the prearcuate region, by using a combination of cyto-, myelo-, and chemoarchitectonic criteria. They identied two areas that are almost completely limited to the anterior bank of the ias, 8/FEF dorsally, and 45B ventrally, and two other areas occupying the ventral prearcuate convexity, area 8r, rostral to area 8/FEF, and area 45A, rostral to 45B.

In this study, based on coronal sections, the prearcuate region was subdivided into three areas, 8d dorsally, 45 ventrally, and 8v between them. Dorsally, a transitional region between areas 9d and the agranular cortex of the PcG was observed; however, this region was not consistently characterized as an architectonically independent area. Functional studies indicate that, in *Cebus *as well as in *Macaca*, the region designated as area 8d coincided with the frontal eye field [40-46].

The map presented in this study also differed from Walker's descriptions [20] regarding the precise localization of area 46 and its borders with areas 9 and 12. This region has been thoroughly studied in primates because of its main role in complex cognitive processes related to the working memory [6-10,47,48] and also because of its possible recent phylogenetic origin [5]. In his study, Walker [20] describes area 46 as extending dorsally and ventrally in relation to the prs, occupying part of the MFG and IFG. More recent studies on *Macaca *diverged in defining this area. While Barbas and Pandya's [25] findings are in general agreement with Walker's map, Preuss and Goldman-Rakic [5] (Figure 1D) recognized areas 46d and 46v in the walls of the prs and areas 46dr and 46vr in the dorsal and ventral rims of the prs respectively, a division compatible with our observations in *Cebus*. Likewise, Petrides and Pandya [28,37] confined area 46 to the lips of the rostral extent of the prs, while they designate the cortex on the lips of the caudal portion of the prs and the immediately adjacent cortex as area 9/46, indicating that this area had been included as part of area 9 in the classic maps of the human cortex.

Cytoarchitectonically, the *Cebus *OfC presented a progressive differentiation from a homotypical granular cortex near the frontal pole to an agranular pattern in the caudal region, a characteristic also observed in *Macaca *[13,26]. Some of the architectonic tools used in this study show a similar transition from rostral to caudal. Rostral areas for example, have an extremely weak WFA staining near the frontal pole becoming more intensely stained caudally.

In *Cebus*, von Bonin [32] recognized only two areas in the OfC, the orbital extension of the "frontal granular anterior area" and the "frontal orbital area", both having several common architectonic characteristics. In the present parcellation, the OfC was divided into five different areas. Although the same designation used by Walker [20] was adopted, some areas of the *Cebus *OfC were subdivided and the limits changed due to their heterogeneity. These findings are in accordance with recent studies on *Macaca*. According to Carmichael and Price [27], in *Macaca *the medial orbital sulcus (mos) also divides area 13 into medial (areas 13b and 13a) and lateral (area 13m) areas (Figure 1C). In *Cebus*, the medial sector of area 13 (13m), lateral to area 14, seems to partly correspond to area 13a described by Amaral and Price in *Macaca *[24], areas 13a and b described by Carmichael and Price [27] (Figure 1C), and was probably included in area 14 (14L, 14VL) by Preuss and Goldman-Rakic [5] (Figure 1D).

The present division of area 12 into two areas (12o and 12l) is in line with the cyto- and myeloarchitectonic divisions proposed by Barbas and Pandya [25] and Preuss and Goldman-Rakic [5] in *Macaca*. Petrides and Pandya [29] designated this area as 12/47, in order to standardize the human and monkey architectonic characteristics. Thus, indicating that the previously labeled area 47 in the human brain is similar in architecture to Walker's area 12.

In *Cebus*, area 14 broadly coincided with the GRe, and consisted of rostral (14r) and caudal (14c) areas. This basic parcellation is largely in agreement with previous studies on the *Macaca *PfC that described the GRe as consisting of at least one rostral and one caudal sector, such as areas 14 and 25 of Barbas and Pandya [25]; 14r and 14c of Carmichael and Price [27], and areas 14a, 14l, 14vl, 14v, and 14 m of Preuss and Goldman-Rakic [5].

Caudally, in the immediate vicinity of the anterior olfactory nucleus and the prepiriform cortex, the OfC assumed a clear agranular aspect. In macaque monkeys, these agranular-periallocortical areas (Oa-p and O-Ins, Figure 3) that correspond to the caudal continuation of areas 12 and 13, had received different designations. Many authors have associated this region of the primate cortex with the insula and the claustrum [49].

In the medial region of the PfC, the present parcellation was consistent with previous studies on *Macaca*, especially with the maps from Vogt et al. [36] and Carmichael and Price [27], which recognized the medial projections of areas 9, 10, 14 and the limbic areas 32, 25 and 24 in the medial wall of the frontal lobe. In *Cebus*, the "limbic anterior area" described by von Bonin [32] was subdivided into areas 32r, 32c and 25. Area 32c, rostral to the corpus callosum, partly corresponds to the macaque area 32 of Barbas and Pandya [25], Vogt et al. [39] and Carmichael and Price [27] (Figure 1C), and area PL of Preuss and Goldman-Rakic [5]. Dorsal and ventrally, this area was separated from superior and inferior adjacent cortical regions by clear-cut boundaries of WGA and SMI-32 staining intensity. Rostrally, *Cebus*'s area 32r seemed to correspond with area MF of Preuss and Goldman-Rakic [5].

Area 25, ventral to area 32c, resembled the one of equal designation described by Vogt et al. [36], Carmichael and Price [27] and Barbas and Pandya [25] in *Macaca*, as well as area IL of Preuss and Goldman-Rakic [5].

### Validity of areas and functional implications

Due to the lack of more detailed information about the connectivity and function of the *Cebus *PfC, it is difficult to know if the areas revealed in this study correspond to functional cortical areas. Several studies, however, indicate that some of the architectonic tools used in this investigation are in fact able to accurately identify brain morphofunctional areas and their boundaries.

WFA staining, for example, has been successfully used to define functional cortical areas in marsupials [50], rats [51,52], mice [53] and Mongolian gerbils [54]. Furthermore, WFA staining also has an area-specific distribution pattern within the human visual, motor and somatosensory cortices [55-57], and thalamus [58].

Similarly, SMI-32 has been used as a powerful tool to reliably define architectonic limits between functional cortical areas in rodents [59], cats [60] and primates [27,61-69]. In addition, SMI-32 has been able to successfully distinguish the primary and middle temporal cortical visual areas of *Cebus *monkey [70].

The functional significance of the heterogeneous distribution of some of the probes used in this study throughout the *Cebus *PfC is not completely known. SMI-32 labels a subpopulation of pyramidal neurons in the primate cerebral cortex [71], and other neuronal types in the thalamus and cerebellum [70]. Based on these results, it is possible to conclude that SMI-32 can identify neurofilament components in neuronal populations with different morphological, functional and connectional characteristics [60].

In the cerebral cortex, SMI-32 positive neurons are mainly located in layers III and V, but depending on the cortical area, the proportion of these cells in each layer may vary. It is known that layer III is the main source of ipsi- and contralateral corticocortical projections and layers V and VI are preferentially associated with subcortical targets [72]. The larger number of positive SMI-32 neurons in layer III, observed in the present study, seems to indicate that this probe preferentially labels corticocortical projection cells in *Cebus *PfC.

Regarding WFA, the extracellular matrix (EM) and PNs have been associated with stabilization and formation of synapses, guiding of axons to their targets, maintenance of the composition of the extracellular compartment, formation of a link with the intracellular cytoskeleton, and concentration of growth factors surrounding certain neurons [73]. These functions attributed to EM and PNs might potentially modify local neuronal activity and thus contribute to the functioning of neuronal networks. Additionally, the fact that PNs were initially observed surrounding GABAergic, fast-spiking non-pyramidal neurons has led some authors to suggest that PNs are mainly involved in local inhibitory circuits [74,75]. However, the present results and those obtained in other studies analyzing different species and cortical areas [76] indicate that a significant amount of pyramidal neurons, mainly in infragranular layers, have a dense covering of PNs (Figure 5E,F). This fact might indicate a relationship between PNs and corticofugal excitatory circuits.

## Conclusions

This study indicated the existence of structural similarities between the *Cebus *and *Macaca *PfC. Cortical areas, such as area 46 on the DlPfC [5,9], considered evolutionary specializations of anthropoid primates were identified in the *Cebus *PfC, based on their topographical and architectonic characteristics. Additional information on the connectivity, chemical structure (in progress at our laboratory), and function of the *Cebus *PfC could clarify how these phylogenetically recent cortical areas have responded, from an evolutionary and adaptative perspective, to the different environmental pressures faced by New and Old World monkeys during the 35 millions of years of parallel evolution.

## Methods

For this study, five young adult male *Cebus apella *monkeys obtained from the Primate Center at the School of Dentistry of Araçatuba (UNESP - Univ Estadual Paulista, São Paulo, Brazil) were used. Experimental procedures were conducted according to the *Guidelines for the care and use of mammals in neuroscience and behavioral research *[77] and were approved by the local laboratory animal care and use committee (Comissão de Ética na Experimentação Animal - CEEA-FOA/UNESP # 2007-002476). All efforts were made to reduce the number of animals and to minimize suffering.

Animals were anesthetized with sodium pentobarbital (30 mg/kg, i.p.) and transcardially perfused with 0.9% saline (800 ml) followed by 1500 ml of 4% paraformaldehyde in 0.1 M acetate buffer, pH 6.5, and subsequently by 1500 ml of 4% paraformaldehyde in 0.1 M borate buffer, pH 9.0. Brains were exposed and blocked with the aid of a stereotaxic device. Blocks were then removed from the skull and placed in a cryoprotective solution containing 10% glycerol and 2% dimethyl sulfoxide in 0.1 M borate buffer, pH 9.0, at 4°C. Three days later, blocks were transferred to a similar solution but with increased concentration of glycerol (20%) for four additional days according to previously described methods [78]. To avoid formation of crystals that may occur during the freezing of large brain pieces, the blocks were immersed in isopentane at -80ºC for one hour to allow quick freezing and then sectioned at 40 μm on the coronal plane using a freezing microtome (SMR 2000, Leica Instruments, GMbH, Germany) and dry ice. Sections were collected in ten different series in a solution of 0.1 M phosphate buffer, pH 7.3.

### Staining methods

#### WFA histochemistry

Free-floating sections were treated with 1% H2O2 in 0.1 M Tris-buffered saline (TBS) for 30 min, washed and subsequently incubated with 2% bovine serum albumin (BSA) in TBS for 1 h. Following three rinses in TBS, sections were incubated with biotinylated WFA (Sigma, L1766) at a concentration of 3 µg/ml TBS-BSA for 16 h, gently shaken at 4°C. The sections were then rinsed in TBS and incubated for 1 h in Extravidin-Peroxidase (Sigma, E2886). Lectin-binding sites were visualized with the chromogen VIP (Vector, SK4600) which yielded a red-purple reaction product. Sections were then mounted on gelatinized slides, dehydrated through a graded alcohol series (70-90-100-100%, 1 min each), cleared in xylene (three changes, 5, 10, and 30 min each) and coverslipped with DPX. In control experiments, biotinylated WFA was omitted and no specific staining was observed in these sections.

#### Black Gold II staining

Sections were previously mounted on 1% gelatin-coated slides and air dried, rehydrated in distilled water for 2 min and transferred to a 0,2% Black-Gold solution at 60°C, for 12-18 min. This solution was made by adding 100 mg of Black-Gold II (Histo-Chem Inc. # 1BGII) to 50 ml of 0.9% NaCl. Incubation was interrupted when the horizontal parallel fibers of layer I were visible. Sections were rinsed for 2 min in distilled water, fixed for 3 min in a sodium thiosulfate solution, rinsed in tap water for 10 min (two 5 min changes), dehydrated through a graded alcohol series (70-90-100-100%, 1 min each), cleared in xylene (5, 10, and 30 min) and coverslipped with DPX.

#### SMI-32 immunohistochemistry

Free-floating sections were treated with 0.6% H2O2 and 60% methanol in TBS 0.1 M for 30 min, washed and subsequently incubated with 3% fetal calf serum (FCS) in TBS for 1 h. Following three rinses in TBS, sections were incubated in a solution of mouse monoclonal SMI-32 antibody (Sternberger Monoclonals, Inc., dilution 1:4000) in TBS-TX 0.05M, for 48 h, gently shaken at 4°C. The sections were then rinsed with TBS-TX for 30 min and incubated for 2 h at 4°C in biotinylated secondary goat anti-mouse antibody (1:200, Vector Laboratories), rinsed again (3 × 10 min TBS-TX) and reacted for 90 min in the Vectastain ABC Elite system (1:100,Vector Laboratories) at room temperature. Antigenic sites were visualized with 0.02-0.05% 3,3'-diaminobenzidine tetrahydrochloride (DAB) and 0.1% nickel ammonium sulfate. Sections were then mounted on gelatinized slides, dehydrated through a graded alcohol series (70-90-100-100%, 1 min each), cleared in xylene (5, 10, and 30 min) and coverslipped with DPX. In control experiments, the SMI-32 antibody was omitted and no specific staining was observed.

#### Nissl stain

The conventional thionin staining method was used to establish the general cytoarchitectonic characteristics and to aid the localization of the laminar distribution of the other stains.

### Analysis

Sections were examined by brightfield microscopy. Selected images were digitalized at high and low magnifications using a Leitz Aristoplan microscope or a Carl Zeiss stereomicroscope (STEMI 2000-c) respectively, both coupled with a Carl Zeiss Axiocam MRc5 digital camera. To eliminate the background originated by digitalization, color balances, brightness, contrast and sharpness were corrected in each preparation.

Because the distinction of limits between areas may vary among observers, the sections were independently examined by three of the researchers of this study and, when necessary, a consensual border was adopted. As defined in previous studies using similar multiarchitectonic approaches, the areas were defined only if they showed differential staining patterns in at least two morphological methods and if they could be consistently found in all animals studied [35].

Two-dimensional schematic representations of the lateral, orbital and medial surfaces of the *Cebus *PfC were designed (Figure 4), showing the approximate location of areal boundaries, as presented in previous studies (Figure 1).

The designation terms of sulcus and gyrus for *Cebus *monkeys used by von Bonin [32], the Template Atlas of the Primate Brain [79], and A stereotaxic atlas of the brain of the *Cebus *monkey (*Cebus apella*) [80] were adopted in this study.

## List of abbreviations

ACgG: anterior cingulate gyrus; as: arcuate sulcus; cgs: cingulate sulcus; DlPfC: dorsolateral PfC; FOG: fronto-orbital gyrus; Gre: gyrus rectus; IFG: inferior frontal gyrus; IG: indisium griseum; iras: inferior ramus of arcuate sulcus; los: lateral orbital sulcus; LorG: lateral orbital gyrus; MFG: medial frontal gyrus; MOrG: medial orbital gyrus; mos: medial orbital sulcus; MPfC: medial prefrontal cortex; Oa-p: periallocortical division of the agranular orbital cortex; OfC: orbitofrontal cortex; O-Ins: orbito-insular cortex; PfC: prefrontal cortex; PrCO: precentral opercular cortex; PrG: precentral gyrus; prs: principal sulcus; ros: rostral sulcus; SFG: superior frontal gyrus; sras: superior ramus of arcuate sulcus; WFA: *Wisteria floribunda *agglutinin

## Authors' contributions

RJC-R designed the study and drafted the manuscript. RJC-R and MAXL carried out the histological processing, microscopic analysis, data collection and preparation of figures. CAC, JAO e EE contributed to microscopic analysis and additional experimental procedures.

All authors read and approved the final manuscript.
